# Orthoplastic Surgery: A Shining Example for ‘Collaborate and Flourish’ in Multidisciplinary Care

**DOI:** 10.1055/s-0045-1812317

**Published:** 2025-10-31

**Authors:** Hari Venkatramani

**Affiliations:** 1 Department of Plastic Surgery, Hand & Reconstructive Microsurgery and Burns, Ganga Hospital, Coimbatore, Tamil Nadu, India

*“In the long history of humankind, those who learned to collaborate and improvise most effectively have prevailed.”*

- *Charles Darwin*

For centuries, “Life before limb,” has been the guiding principle in trauma care. Even during the Second World War, a major open fracture with skin and soft tissue loss was considered a life-threatening condition, because the perceived risks to life were enormous and the chances of limb salvage was small. Infection was the main cause of morbidity and mortality, and the inability to cover major gaps in tissues was the cause for the low success rates of limb salvage. The advent of microsurgery made it possible to cover large gaps in both soft tissues and bone. At about the same time, encouraged by the newfound capability of covering large soft tissue gaps, surgeons performed radical debridement. Infection rates dropped, and a new chapter in trauma reconstructive surgery was thus born.

Besides techniques, what really caused the dramatic improvement was the synergistic alliance between plastic and orthopaedic surgeons, which was aimed at optimizing outcomes in limb reconstruction and this discipline was called “orthoplastic surgery.” 1 Bratton and Tumin once proposed the stark dictum “collaborate or perish,” but in modern practice this has evolved into a more optimistic adage: “collaborate and flourish.” 2 Credit for this alliance must be given to institutions like the Kleinert Institute in Louisville, United States, where surgeons from orthopaedic, plastic, and general surgical backgrounds all worked together. Each did what they could do best, and everyone sought the help of the other if needed, and ultimately, the patient had good outcomes. Some names who have to be honored are Marko Godina, a Slovenian surgeon who was a fellow in Louisville in 1978. He went back to his war-ravaged country and had an opportunity to introduce the early soft tissue cover for open fractures. A few years later, he came back to Louisville and presented his enormous experience. The Kleinert team encouraged him to write it up. He stayed for a few weeks and wrote the draft. Unfortunately, on return he was killed in a tragic road traffic accident in 1986 at the age of 43. Lister and Kleinert took the responsibility of translating his work from Slovenian to English and wrote up a limited edition called “The Thesis.” 3

Godina stressed that the cause for the poor outcome was the artificial separation of the two specialties, orthopaedic and plastic surgery, which made them see the patient sequentially and not together. The system of orthopaedic and plastic surgeons, simultaneously seeing the patient, gradually became the recommended standard of care. Scott Levin, who was a fellow in Louisville in 1986, is credited for coining the term “orthoplastic surgery.”

Recently, an international journal has been started called “ *Orthoplastic Surgery* ” to promote advances in this discipline. An International Congress for Orthoplastic Surgeons is going to be held in New York from May 14 to 16, 2026. 4

## What is the Status of Orthoplastic Surgery in India?

Until the early nineties of the last century, there was very little collaboration between orthopaedic and plastic surgeons. Raja Sabapathy who was trained at Louisville and Canniesburn in the late eighties gave a big push for this field. Together with his brother Rajasekaran (orthopaedic surgeon), they made orthoplastic surgery a norm at Ganga Hospital, Coimbatore. The paper on “Demanding Plastic Surgical Procedures in Acute Limb Trauma,” which was awarded President's Special Peet Prize in the 1993 Bangalore APSICON, was one of the earliest papers presented on collaborative approach in APSICON. The Ganga Hospital Open Injury Severity Score (GHOISS) for open tibial fractures stands as a seminal contribution born of this orthoplastic collaboration and is accepted the world over. 5

In 1998, Raja Sabapathy conducted a survey on the “Availability and Utilization of Plastic Surgeons in the Management of Open Fractures in India,” under the Venture Fund of Association of Plastic Surgeons of India (APSI). The study was conducted by sending questionnaires to all the members of the APSI and Indian Orthopaedic Association (IOA). A total of 406 orthopaedic surgeons and 163 plastic surgeons participated. The study was published in 1999 in the *Indian Journal of Plastic Surgery* and throws up some very interesting findings. The study revealed that 36% of plastic surgeons were not involved in managing open fractures, often due to lack of referrals, poor infrastructure, or limited interest in trauma. On the orthopaedic side, only 9% involved plastic surgeons in all cases, while nearly half were trained without exposure to interdepartmental collaboration. Although published a quarter of a century ago, this study remains strikingly relevant, as many of the highlighted obstacles—delayed referrals, inadequate training exposure, and interdepartmental egos—persist to this day. I would think that if the study is to be repeated the results may not be very different. 6

## What Solutions Do We Have Which Will Help Us Break the Barriers?

Looking ahead, the way forward lies in embedding collaboration into training, policy, and practice. Mandatory cross-training between orthopaedics and plastic surgery, hospital protocols requiring early joint evaluation with senior input at the onset, regional orthoplastic referral networks, and leadership-driven incentives for teamwork can transform intent into reality. By aligning education, systems, and culture, orthoplastic care can evolve from an isolated success in select centers to a national standard of trauma management.

Looking back on my own personal journey of three decades after completing my specialization in plastic surgery from Mumbai, I joined Ganga Hospital in 1999. I like to call myself a trauma reconstructive surgeon who managed complex wounds and have built my practice around it. By concentrating on this area, we refined the existing techniques, pushed the boundaries of salvage, and produced better outcomes. Even today, when on call I like to be the first person who sees the patient to help make the important decisions. Scott Levin once said that “a day without microsurgery is a day without sunshine,” I would rephrase it as “a day without making a difference in the life of someone is a day wasted.”

In 2019, as the guest editor of the special issue on lower limb reconstruction of the *Indian Journal of Plastic Surgery* , we had asked this question that why no one styles themselves as a lower limb reconstructive surgeon. 7 At present, there are no fellowships in this field in India, nor any dedicated subspecialty association. However, the scope of orthoplastic surgery is rapidly expanding beyond its initial focus on just managing traumatic limb reconstruction to encompass diabetic foot reconstruction, musculoskeletal oncology, and congenital deformity correction. There is a huge unmet demand for orthoplastic collaboration in India.

## Orthoplastic Principles as Applicable Today

The discipline is not just a sequence of steps but more a philosophy of joint ownership from the moment the patient arrives.

Joint evaluation: On arrival, the orthopaedic and plastic surgeons, working side by side, assess the limb under regional anesthesia. 8 This immediate collaboration avoids delay, prevents infection, and ensures resources are used wisely. Anesthesiologists complete this triad, stabilizing the patient so that surgical plans are not built on a fragile foundation.

Joint decisions: The question of salvage versus amputation is the most important question for the patient, and it cannot be answered by either specialty alone. Using structured tools such as the GHOISS, 9 the further plan of action is decided taking the patient and family in confidence. Amputation, when chosen, is a reconstructive path and salvage when pursued must deliver function equal to or better than prosthesis, which is the ultimate goal. 5

Joint action: Patient is taken up for debridement, which involves clearing the field of contaminants and contused hypovascular tissues while preserving the uninjured longitudinal structures like tendons, nerves, and vessels. Temporary external fixation provides stability, while early flap coverage protects bone and vessels, following Godina's timeless dictum. Since the soft tissue cover is assured, the orthopaedic surgeon could choose the best possible fixation for the fracture.

Now let us see how we put the principles of orthoplastic surgery in practice through an actual case where the principles combined to salvage a limb.

## Case Study

A 20-year-old male sustained a devastating crush injury after falling from a train in 2016. Initial treatment was taken at a local hospital where a right below-elbow amputation and a left below-knee amputation were done. The right lower limb, also severely injured, was considered unsalvageable. At that point, he stood on the brink of becoming a triple amputee.

He was brought to Ganga Hospital, and what followed was a 2-year journey of staged and coordinated care. He was initially treated with radical debridement and external fixation, which was followed by a microvascular latissimus dorsi free flap by Godina approach to restore coverage. Distraction osteogenesis was done later for the tibial defect reconstruction.

**Fig. 1:**
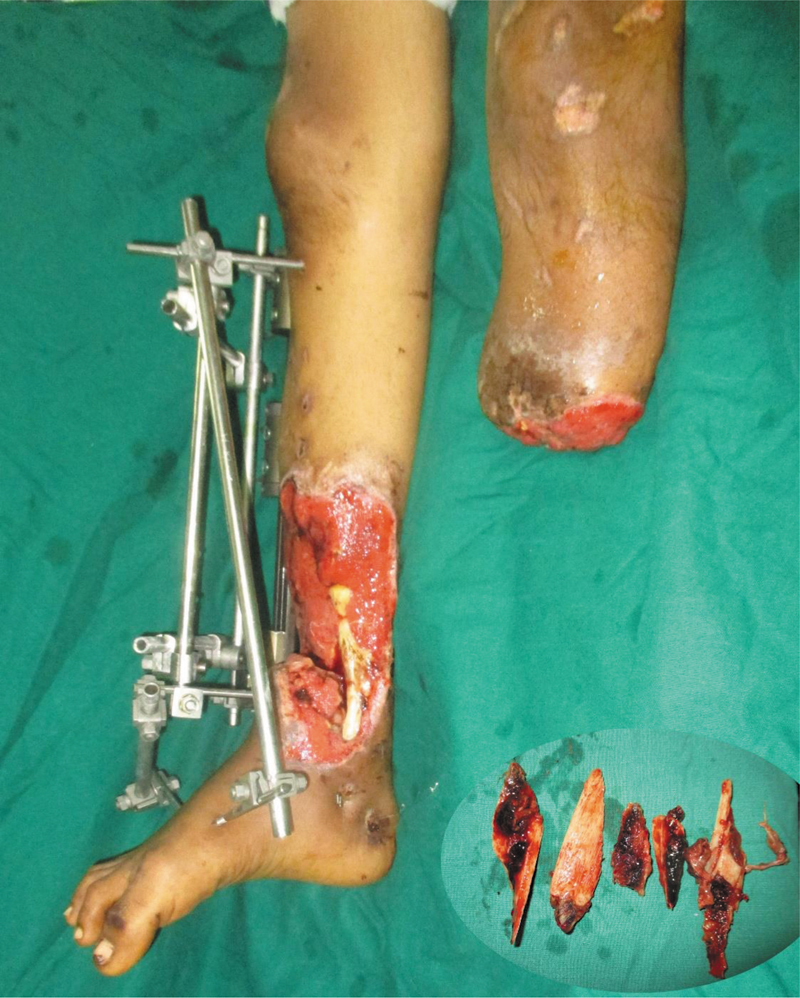
Type 3B open fracture of right lower limb (inset: loose bone fragments removed), left below knee amputation.

**Fig. 2:**
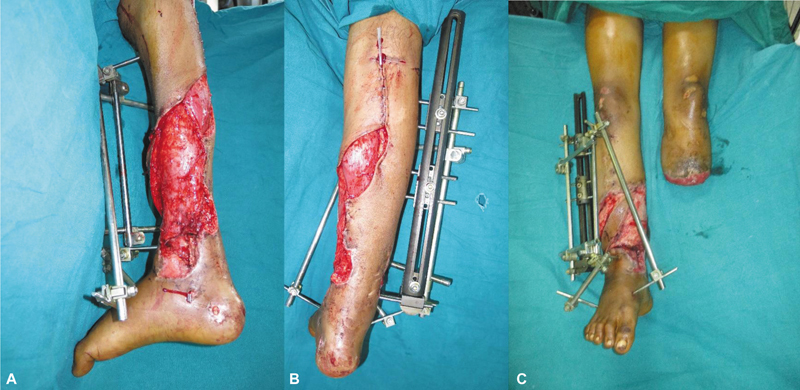
( **A** – **C** ) Contralateral free latissimus dorsi muscle flap anastomosed to injured posterior tibial vessels through Godina's approach.

**Fig. 3:**
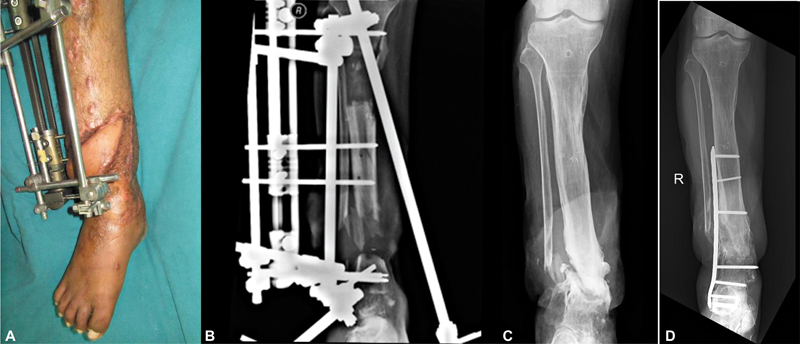
( **A** ) Well-settled muscle flap. ( **B** ) Bone transport in progress. ( **C** ) Completed bone transport. ( **D** ) Nonunion treated by plating and bone grafting.

**Fig. 4:**
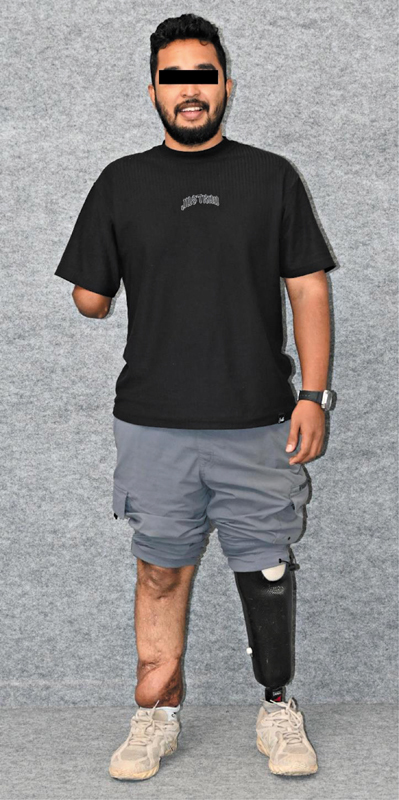
Well-settled flap with length of limb restored, the opposite limb fit with a transtibial prosthesis.

The patient uses the reconstructed limb to propel himself forward. Each step reflected the essence of collaboration, and together, these efforts preserved one lower limb that otherwise would have been lost Figs. 1 to 4 .

## Advantages and Future Directions of Collaboration

The benefits of orthoplastic collaboration have been well established. When orthopaedic and plastic surgeons work with total trust in each other, each specialty strengthens the other—bone reconstruction inspires more daring flap design, while innovative coverage challenges fixation to evolve. This mutual pulling-up effect creates a cycle of progress that no department could achieve alone. The outcome is not only higher salvage rates but also limbs that are both functional and esthetically restored, reducing morbidity and elevating quality of life.

The future of orthoplastic surgery will be written at the intersection of trust and technology, guided by leadership. Telemedicine and multicenter collaborations can democratize access to orthoplastic expertise. Artificial intelligence may soon predict outcomes and guide choices in limb salvage.

The ethos of “collaborate and flourish” is now echoed beyond limb trauma. Oncoplastic breast surgery merges tumor clearance with esthetics; oculoplastic surgery restores both vision and form; craniofacial surgery integrates plastic surgery, orthodontics, and speech; and burn care unites plastic surgery, intensive care, and rehabilitation. The important point to remember is that a collaboration succeeds when we give more than what we expect from other.

The Nobel Laureate and the first ever transplant surgeon, Joseph Murray, who was also a plastic surgeon once said, “It is in the interface of the specialities that progress is made.” Orthoplastic surgery, thus, is more than the sum of orthopaedics and plastic surgery and stands as a living embodiment of the new dictum “collaborate and flourish.”
